# Altitude control in honeybees: joint vision-based learning and guidance

**DOI:** 10.1038/s41598-017-09112-5

**Published:** 2017-08-23

**Authors:** Geoffrey Portelli, Julien R. Serres, Franck Ruffier

**Affiliations:** 1 Aix Marseille Univ, CNRS, ISM, Marseille, France; 2 Université Côté d’Azur, CNRS, I3S, Sophia Antipolis, France

## Abstract

Studies on insects’ visual guidance systems have shed little light on how learning contributes to insects’ altitude control system. In this study, honeybees were trained to fly along a double-roofed tunnel after entering it near either the ceiling or the floor of the tunnel. The honeybees trained to hug the ceiling therefore encountered a sudden change in the tunnel configuration midways: i.e. a "dorsal ditch". Thus, the trained honeybees met a sudden increase in the distance to the ceiling, corresponding to a sudden strong change in the visual cues available in their dorsal field of view. Honeybees reacted by rising quickly and hugging the new, higher ceiling, keeping a similar forward speed, distance to the ceiling and dorsal optic flow to those observed during the training step; whereas bees trained to follow the floor kept on following the floor regardless of the change in the ceiling height. When trained honeybees entered the tunnel via the other entry (the lower or upper entry) to that used during the training step, they quickly changed their altitude and hugged the surface they had previously learned to follow. These findings clearly show that trained honeybees control their altitude based on visual cues memorized during training. The memorized visual cues generated by the surfaces followed form a complex optic flow pattern: trained honeybees may attempt to match the visual cues they perceive with this memorized optic flow pattern by controlling their altitude.

## Introduction

In the general framework of altitude control, the visual stimuli encountered in a tunnel consist of the optic flow (OF) vector field (its density, magnitude and/or direction) and the retinal positions of any contrasts (patterns and/or tunnel edges) perceived. Insects are known to sense the direction and angular velocity of the OF provided by the contrasting features in the environment crossing their retina and use them to control their flight^1–10^. The OF vector field perceived by a moving animal depends on the structure of the environment^11, 12^. The magnitude of the *translational* OF, which describes the front-to-back motion occurring on the retina when the insect is moving forward, depends on the ratio between the insect’s relative speed and the distance from the contrasting objects in the environment. In *Drosophila melanogaster* fly, the displacement of horizontal contrasting edges and the divergent OF significantly affect the insects’ altitude^9^. Trained honeybees have been found to make significant changes in their altitude in response to OF cues perceived in the ventral regions of the eyes, depending on the strength (or density) of the longitudinal OF cues (axial stripes forming weak cues, versus chequerboard patterns forming strong cues)^7^ and on the magnitude of the OF, which was varied by changing the speed of the tunnel floor^8^.

Honeybees flying along a wide corridor do not systematically follow the midline of the corridor but tend rather to hug one wall, namely that nearest to the position of the entrance used during the training step^13^. Honeybees have also been found to follow one wall by maintaining the unilateral OF constant^13^ and to follow the floor by maintaining the ventral OF constant^5, 8^. Trained honeybees have been generally found to keep the OF generated by the floor at a similar value to that experienced during the training step^8^. It has been suggested that the use of an *optic flow regulator* may explain how flying insects take off, cruise, react to wind, and land at a constant slope on the sole basis of the OF they perceive ventrally^14^.

In the ALIS model^15^, this concept of downward *OF regulation* was extended to all 3 dimensions^14, 16, 17^, and the concept of *dual OF regulation* was also applied in both the vertical^18^ and horizontal planes^19^). The ALIS model suggests that the OF perceived in the lateral, ventral and dorsal regions of the eyes are all involved in honeybees’ visual guidance processes, as subsequently found to occur in behavioural studies^20^. The biorobotic-inspired learning-free ALIS model predicted, for example, that a honeybee flying in a high-roofed tunnel will follow the surface nearest to the position of the entrance, even if that surface is the ceiling.

As a fair return to biology, the first question addressed here is whether honeybees are able to follow the ceiling of a high-roofed tunnel in the same way as they follow a wall^13^ or the floor^8^. The second question which arises is whether and how honeybees memorize the surface followed during a training step in which they learn to retrieve a reward. With a view to answering these questions, honeybees were trained to fly along a high-roofed tunnel, and their behavioural responses to a sudden change in the tunnel height (which we have called “dorsal ditch” conditions), causing a strong dorsal OF perturbation, were then observed. Honeybees’ surface-following behaviour was also tested by changing the vertical position of the entrance to the tunnel with respect to the positions of the entrances used under training and control conditions. Honeybees’ trajectories were recorded and special attention was paid to the way the insects responded to the sudden changes in tunnel height generating an OF disturbance, and how they adjusted their vertical position in the tunnel depending on the vertical position of the entrance to the tunnel.

## Results

**Figure 1:**
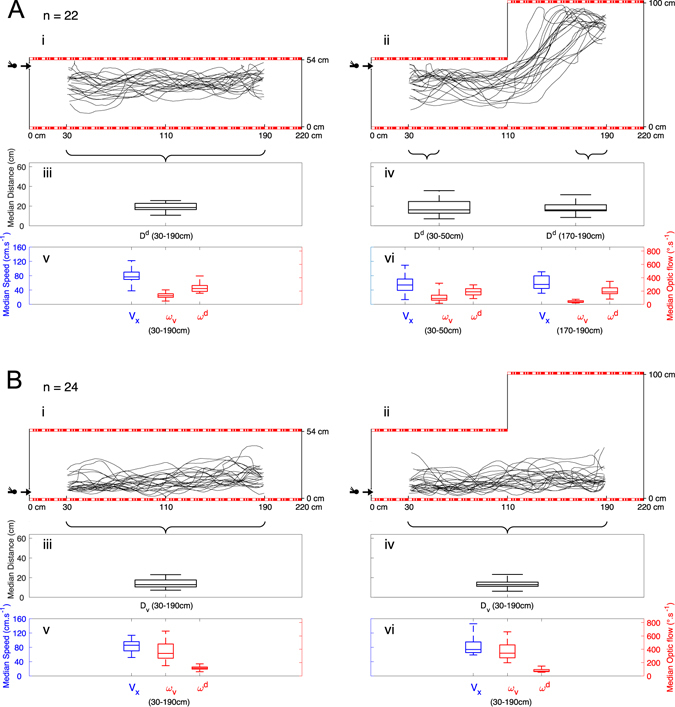
(**A**) Step 0: *E* ^*Top*^ → *R* ^*Top*^; Steps i&ii: *E* ^*Top*^ Trajectories of honeybees (*n* = 22) trained to enter the tunnel via the upper entrance before being released at the upper entrance under low-roof conditions, as described in i as well as under “dorsal ditch” conditions, as described in ii. In the flight under “dorsal ditch” conditions which the bees had not previously experienced, the insects flew upward and followed the newly encountered high roof at median distances *D* ^*d*^ (see boxplots in iii and iv) which were not significantly different. Distributions of median speed *v*_*x*_ and median dorsal optic flows *ω* ^*d*^ (see boxplots in v and vi) were also not significantly different each others. (**B**) Step 0: *E*_*Bottom*_ → *R*_*Bottom*_; Steps i&ii: *E*_*Bottom*_. Trajectories of honeybees (*n* = 24) trained to enter the tunnel via the lower entrance before being released at the lower entrance under low-roof conditions, as described in i and again via the lower entrance under “dorsal ditch” conditions, as described in ii. In the flight under “dorsal ditch” conditions which the bees had not previously experienced, the insects flew near the floor at median distances *D*_*v*_ (see boxplots in iii and iv) which were not significantly different. Distributions of median speed *v*_*x*_ and median ventral optic flow *ω*_*v*_ (see boxplots in v and vi) were also not significantly different each others. (See statistics in results and discussion sections).

Twenty-two individual honeybees were trained to enter the tunnel near the ceiling in Fig. 1Ai under constant low-roof conditions, before the inner roof was removed and then released via the same entrance under “dorsal ditch” conditions, as shown in Fig. 1Aii. Interestingly, the honeybees trained to enter the tunnel near the ceiling changed their flight height when flying below the “dorsal ditch” (Fig. 1Aii). This 2-step experiment was repeated for twenty-four other individual honeybees trained to enter the tunnel near the floor (Fig. 1Bi) and then released via the same entrance under “dorsal ditch” conditions (Fig. 1Bii): the honeybees trained to enter near the floor kept following the floor under the “dorsal ditch” (Fig. 1Bii).

### The position of the entrance during training determined the honeybees’ vertical position

When honeybees were trained to enter near the ceiling of the tunnel (*n* = 22) (Fig. 1Ai), they flew along the tunnel at a height of 35.4 ± 4.0 cm (Median ± MAD -Median Absolute Deviation-), following the ceiling therefore at a distance *D* ^*d*^ of 18.6 ± 4.0 cm (Fig. 1Aiii). When honeybees were trained to enter near the floor of the tunnel (*n* = 24) (Fig. 1Bi), they flew along the tunnel at a height (here *D*_*v*_) of 12.9 ± 3.2 cm (Fig. 1Biii). The position of the entrance during the training step therefore significantly affected the honeybees’ flight height (one-tailed *two-sample Wilcoxon*-test, *W* = 528, *P* < 0.001), which was greater in the recording shown in Fig. 1Ai than in that shown in Fig. 1Bi. The flight height observed depended on the vertical position of the entrance used during the training step.

### Trained honeybees kept following the surface in the same direction and at a similar distance in the vertical plane

Immediately after recording the bees’ flight paths under low-roof conditions, the inner roof was removed and the flight was repeated under “dorsal ditch” conditions, as shown in Fig. 1Aii and Bii.

Interestingly, the honeybees trained to enter the tunnel near the ceiling changed their flight height while flying below the “dorsal ditch” (Fig. 1Aii). In the first part of the tunnel (steady state between *x* = 30 cm to *x* = 50 cm), the honeybees flew at a height of 37.9 ± 5.6 cm (Fig. 1Aiv). In the second part (steady state between *x* = 170 cm to *x* = 190 cm), they flew at a height of 83.8 ± 2.9 cm (Fig. 1Aiv), which was significantly higher than that observed (Fig. 1Aii) in the first part of the tunnel (one-tailed *one-sample Wilcoxon*-test, V = 0, *P* < 0.001). As shown in Fig. 1Ai (in the 30–190 cm range) and Fig. 1Aii (in the 30–50 cm and 170–190 cm ranges), these three distributions of honeybees’ distances from the ceiling showed no significant differences once a steady flight height had been reached (*anova*.*lme*, *F*_2/40_ = 0.1350, *P* = 0.8741) (see also the boxplots in Fig. 1Aiii–iv and the median values in Table 1).

**Table 1:** Horizontal speed *v*_*x*_, height of flight *h* and the dorsal *ω* ^*d*^ and ventral *ω*_*v*_ optic flow (OF) (Median ± MAD -Median Absolute Deviation-) experienced by honeybees during the steady flight height phase while flying along the tunnel in the various experimental conditions.

Training condition	*E* ^*Top*^ → *R* ^*Top*^			*E*_*Bottom*_ → *R*_*Bottom*_	
Entrance position	*E* ^*Top*^			*E*_*Bottom*_	
*Experiments*	Fig. 1Ai	Fig. 1Aii		Fig. 1Bi	Fig. 1Bii
*Tunnel height*	*54* *cm*	*54* *cm*	*100* *cm*	*54* *cm*	*54* *cm and then 100* *cm*
*Steady state Range*	*30–190* *cm*	*30–50* *cm*	*170–190* *cm*	*30–190* *cm*	*30–190* *cm*
*h* (cm)	35.4 ± 4.0	37.9 ± 5.6	83.8 ± 2.9	12.9 ± 3.2	12.9 ± 2.1
*v*_*x*_ (cm/s)	76.8 ± 10.5	54.2 ± 15.9	55.7 ± 17.8	85.8 ± 12.9	73.6 ± 9.4
Dorsal OF *ω* ^*d*^ (°/s)	238.2 ± 43.9	189.0 ± 46.5	186.9 ± 48.8	116.6 ± 17.2	71.6 ± 10.7
Ventral OF *ω*_*v*_ (°/s)	130.1 ± 28.7	88.8 ± 45.4	36.0 ± 10.0	337.7 ± 100.6	341.0 ± 82.5
**Entrance position**	***E*** ^***Top***^	***E***_***Bottom***_		***E***_***Bottom***_	***E*** ^***Top***^
***Experiments***	**Fig. 2Ai**	**Fig. 2Aii**		**Fig. 2Bi**	**Fig. 2Bii**
*Tunnel height*	*54* *cm*	*54* *cm*	*100* *cm*	*54* *cm*	*54* *cm and then 100* *cm*
*Steady state Range*	*30–160* *cm*	*40–50* *cm*	*150–160* *cm*	*30–160* *cm*	*30–160* *cm*
*h* (cm)	35.4 ± 6.0	38.8 ± 5.1	85.6 ± 2.3	12.1 ± 3.8	13.7 ± 3.5
*v*_*x*_ (cm/s)	70.8 ± 11.8	63.6 ± 15.0	41.7 ± 22.0	78.5 ± 9.4	62.3 ± 9.8
Dorsal OF *ω* ^*d*^(°/s)	211.1 ± 57.9	234.85 ± 85.4	163.8 ± 62.2	109.9 ± 21.1	75.8 ± 15.4
Ventral OF *ω*_*v*_(°/s)	110.8 ± 29.3	100.18 ± 29.8	28.6 ± 14.7	374.3 ± 121.1	282.9 ± 57.8

When honeybees had been trained to enter the tunnel near the floor (*n* = 24), they flew under “dorsal ditch” conditions at a height of 14.5 ± 3.7 cm in the second part of the tunnel (steady state between *x* = 110 cm to *x* = 190 cm) (Fig. 1Bii). No significant differences in the flight heights were observed between “constant low roof” condition (see Fig. 1Bi) and “dorsal ditch” condition (between *x* = 30 cm to *x* = 190 cm in Fig. 1Bii) (two-sided *one-sample Wilcoxon*-test, V = 189, *P* = 0.2768).

Trained honeybees kept following the same surface despite the removal of the inner roof, which generated a strong dorsal visual disturbance, and hence regardless of the sudden change in the tunnel’s height.

### Trained honeybees greatly adapted their flight height in order to resume the surface following behavior learned during training in the vertical plane

**Figure 2:**
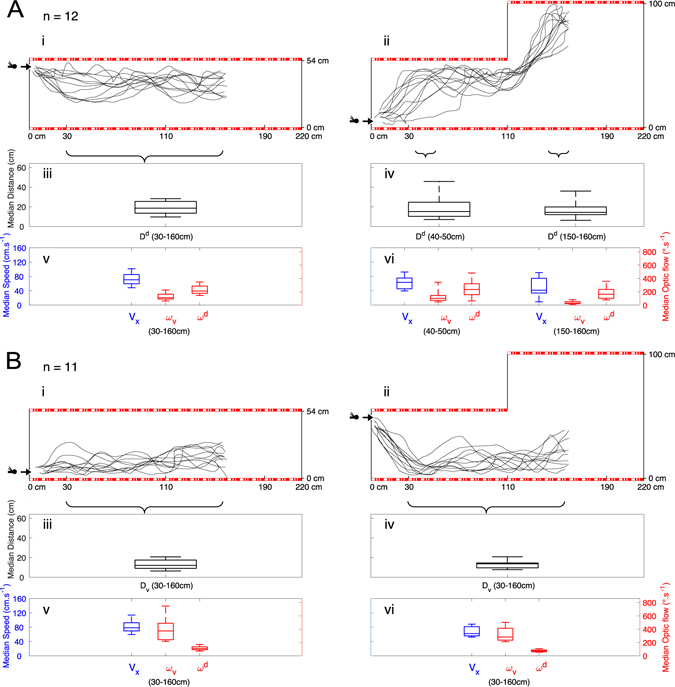
Step 0: *E* ^*Top*^ → *R* ^*Top*^; Step i: *E* ^*Top*^; Step ii: *E*_*Bottom*_. (**A**) Trajectories of honeybees (*n* = 12) trained to enter the tunnel via the upper entrance before being released again via the upper entrance under low-roof conditions, as described in i and then released via the low entrance under “dorsal ditch” conditions, as described in ii. In the flight performed under “dorsal ditch” conditions which they had not previously experienced, the honeybees flew upward from the low entrance and followed the low roof and then continued to follow the newly encountered higher roof at an median distance *D* ^*d*^ (see boxplots in iii and iv) which did not differ significantly from previous condition. Distributions of median speed *v*_*x*_ and median dorsal optic flows *ω* ^*d*^ (see boxplots in v and vi) were also not significantly different each others, when the ascent speed is low. (**B**) Step 0: *E*_*Bottom*_ → *R*_*Bottom*_; Steps i&ii: *E* ^*Top*^. Trajectories of honeybees (*n* = 11) trained to enter the tunnel via the lower entrance before being released again via the lower entrance under low-roof conditions, as described in **i** and then via the upper entrance under “dorsal ditch” conditions, as described in **ii**. In the flight performed under “dorsal ditch” conditions which had not been previously experienced, the honeybees immediately descended and followed the floor regardless of the presence of the dorsal ditch, at median distances *D*_*v*_ (see boxplots in iii and iv) which did not significantly differ from that recorded under low-roof conditions. Distributions of median speed *v*_*x*_ and median ventral optic flow *ω*_*v*_ (see boxplots in v and vi) were also not significantly different each others. (See statistics in results and discussion sections.).

Twelve individual honeybees were trained to enter the tunnel near the ceiling in Fig. 2Ai under constant low-roof conditions, before the inner roof was removed and being released via the other entrance under “dorsal ditch” conditions, as shown in Fig. 2Aii. This 2-step experiment was repeated for eleven other individual honeybees trained to enter the tunnel near the floor (see Fig. 2Bi) and then released via the other entrance under “dorsal ditch” conditions (Fig. 2Bii).

Interestingly, both plots in Fig. 2Aii–Bii show that trained honeybees entering the tunnel via the other entrance changed drastically their flight height under “dorsal ditch” conditions: insects flew closer to the surface followed during the training step under constant low-roof conditions.

As shown in Fig. 2Ai (in the 30–160 cm range) and Fig. 2Aii (in the 40–50 cm and 150–160 cm ranges), the distributions of the distances from the ceiling with and without the inner roof did not differ significantly once the insects reached stable flight pattern, despite the complete change in both the position of the entrance and the height of the tunnel (*anova*.*lme*, *F*_2/22_ = 0.39031, *P* = 0.6814) (see also the boxplots of *D* ^*d*^ in Fig. 2Aiii,iv and median values in Table 1).

Despite the considerable change in both the entrance position and the tunnel height, the distributions of the distances from the floor (flight height) between Fig. 2Bi and Bii did not change significantly (two-sided *one sample Wilcoxon*-test, *V* = 31, *P* = 0.8984) (see also the boxplots of *D*_*v*_ in Fig. 2Biii,iv and median values in Table 1).

All the distances to the nearest surface in the vertical plane recorded in all these experiments (entry via the floor or ceiling) were relatively similar under constant low-roof conditions and under “dorsal ditch” conditions despite the changes in the position of the entrance (*E*_*Bottom*_ or *E* ^*Top*^). Trained honeybees therefore greatly changed their flight height in order to be able to go on using the surface following behaviour they had adopted during the training session, especially in terms of the direction of the memorized visual stimuli encountered in the insects’ field of view.

## Discussion

In the 4 experiments described in this study (Figs 1A,B and 2A,B), honeybees were trained to fly along a tunnel under constant low-roof conditions before being tested in a tunnel with an upward recessed roof, under what we have called “dorsal ditch” conditions, which suddenly perturbed the visual stimuli occurring in their dorsal field of view. Four main points emerged from this study, which will be discussed here.

### In the vertical plane, honeybees followed either the floor or the ceiling

After training honeybees to fly along a wide horizontal corridor, it has been observed that *honeybees do not systematically fly along the corridor midline* ^13^. Honeybees adopted wall-following behaviour whenever both the entrance and the reward source were located near the same wall^13^.

Depending on the entrance position used, honeybees were able not only to follow the ground or the ceiling, but also to systematically keep the surface followed at a similar distance to that previously adopted under constant low roof conditions. The distribution of the distances from the nearest surface showed no significant differences in any of the four experiments between the “low roof” and the “dorsal ditch” conditions (see statistics in the Results section). Based on these findings, honeybees flying along a tunnel can be assumed to proceed by following the surface nearest to the entrance used during training. This finding supports the hypothesis that the visual responses may not differ fundamentally between the dorsal and ventral parts of honeybees’ compound eyes. Trained honeybees may therefore follow the nearest surface in the vertical plane, as similarly found to occur in the horizontal plane^13^.

In a previous study, honeybees were trained to fly in a high-roofed tunnel, part of the floor of which could be set in motion^8^. When honeybees flew over a progressive moving floor (i.e. in the same direction as the bees’ flight), honeybees flew at a lower height by gradually returning to the previously experienced ventral OF. Motion of the floor (*V*_*floor*_ = 0.5 m/s) greatly decreased their ventral OF, which was equivalent to that triggered when flying over a “ventral ditch”.

The present “dorsal ditch” study can be paired with this previously published “progressive moving floor” experiments^8^, (i) resulting first in a similar large decrease, in the dorsal OF and in the ventral OF respectively, and (ii) resulting secondly a similar move toward the followed surface, ceiling and floor respectively. Based on the results of these two studies, it can be concluded that trained honeybees react in the same way to a floor progressive movement that occurs in the ventral viewfield^8^ and to a upside-down ditch that occurs in the dorsal viewfield.

### A preferred OF stimulus is regained in the vertical plane

**Figure 3:**
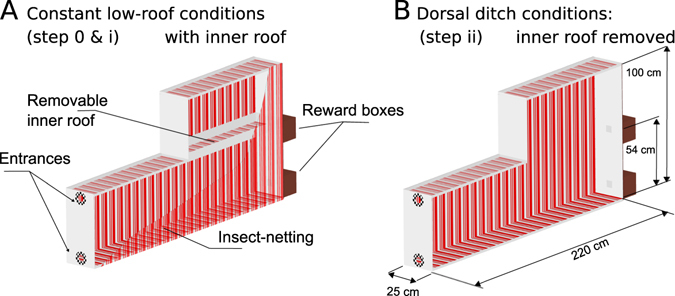
Perspective view of the double-roofed flight tunnel. The floor, roof and left wall of the flight tunnel consisted of planks with red and white stripes oriented transversely to the bees’ flight paths. One side of the tunnel consisted of insect netting lined with stripes formed by a red gelatin filter. The 25 cm-wide and 220 cm-long tunnel consisted of two parts: (i) the first half had a permanent 54-cm low roof, and (ii) the second half had a double roof consisting of a removable inner part set at a height of 54 cm and a permanent upper part set at a height of 1 m. (**A**) Under constant low roof conditions, the inner roof was set at the height (54 cm): the tunnel is therefore endowed with an uniform height of 54 cm. (**B**) Under “dorsal ditch” conditions, this inner roof was removed to make the 1-m high upper roof visible to the honeybees, thus imposing a sudden perturbation on their dorsal visual stimuli. Honeybees were trained to enter the tunnel under constant low roof conditions near either the roof or the floor, depending on the experiment, and they were able to collect a sugar solution reward placed in a box at the end of the tunnel either near the roof or near the floor, accordingly.

In a parallelepipedal tunnel of this kind (a rectangular box, see Fig. 3), several visual cues are theoretically available for use in altitude control processes: (*i*) the vertical angle (or elevation angle) in the field-of-view of the salient parallel lines formed by the corners along this parallelepipedal tunnel (these cues were previously found to be used by ants for lateral positioning purposes^21^), (*ii*) the splay angles created by the geometric perspective between these salient lines (as already addressed in Humans^22^), (*iii*) and the magnitude and direction of the OF vector field (which includes right, left, ventral and dorsal angular speeds). The splay angles and the magnitude of the OF are both visual cues which depend on the distance to the nearest surface of the tunnel.

Flying insects are able to fly along a tubular tunnel^23^ devoid of any salient corners. Trained honeybees were also found to change their flight height^8^ or their lateral position^24^ inside a parallelepipedal tunnel devoid of any sudden changes in height or width, but simply equipped with a horizontally moving floor or wall affecting the magnitude of the translational OF. In the vertical plane, the OFs generated here by the nearest surface of the tunnel were: (i) the dorsal OF shown in Figs 1Ai,ii and 2Ai,ii and (ii) the ventral OF in Figs 1Bi,ii and 2Bi,ii. When the ceiling was the nearest surface present during the training step, the dorsal OF generated did not vary significantly once the insects showed stable flight pattern (data frame: *ω* ^*d*^(Fig. 1Ai 30–190 cm), *ω* ^*d*^(Fig. 1Aii 30–50 cm), *ω* ^*d*^(Fig. 1Aii 170–190 cm), *ω* ^*d*^(Fig. 2Ai 30–160 cm), *ω* ^*d*^(Fig. 2Aii 40–50 cm); *anova*.*lme*: *F*_2/53_ = 2.8622, *P* = 0.0660). When the floor was the nearest surface perceived during the training step, the ventral OF generated by insects once they showed stable flight pattern was not significantly different with respect to two conditions: *constant low roof* condition, *dorsal ditch* condition (data frame: *ω*_*v*_(Fig. 1Bi 30–190 cm), *ω*_*v*_(Fig. 1Bii 30–190 cm), *ω*_*v*_(Fig. 2Bi 30–160 cm), *ω*_*v*_(Fig. 2Bii 30–160 cm); *anova*.*lme*: *F*_1/34_ = 1.2554, *P* = 0.2704).

The honeybees therefore kept the magnitude of either the ventral OF or the dorsal OF constant when the nearest surface available during the training step was the floor or the ceiling, respectively. The present results are in line with those obtained in previous studies^1–8^. In the high-roofed tunnel, honeybees change their flight height in order to recover the familiar OF magnitude previously encountered during training.

### Challenging the validity of the ALIS model

According to the learning-free ALIS model^15^, the flight of a simulated bee is controlled by regulating the visual angular velocity (the magnitude of the translational OF) via two intertwined loops: first, a forward control loop based on the maximum sum of opposite OFs (bi-lateral or bi-vertical; i.e., the maximum sum of the OFs generated by opposite surfaces), and secondly, a positioning control loop based on the maximum OF (right, left, dorsal or ventral; i.e., the maximum OF generated by one surface).

Firstly, the maximum OF sum here was the bilateral OF generated by the two lateral walls of the tunnel, as shown in Figs 1A,B and 2A,B, which can be estimated assuming that honeybees were centred in the horizontal plane (in a narrow tunnel^4^). The distribution of their median flight speeds in 6 experiments in which the honeybees were observed once the insects showed stable and straight flight pattern, was used to assess their likelihoods (data frame: *v*_*x*_(Fig. 1Ai 30–190 cm), *v*_*x*_(Fig. 1Bi 30–190 cm), *v*_*x*_(Fig. 1Bii 30–190 cm), *v*_*x*_(Fig. 2Ai 30–160 cm), *v*_*x*_(Fig. 2Bi 30–160 cm), *v*_*x*_(Fig. 2Bii 30–160 cm); *anova*.*lme*: *F*_1/34_ = 2.3976, *P* = 0.1308).

The ground speed therefore can be said to have been similar in all these tunnels and experiments: this statement may suggest that honeybees may maintain constant their forward speed using a OF bilateral regulator with a forward OF set-point with respect to the minimal cross-section (the lateral one) which is also constant across this tunnel (as previously suggested in^15^).

Secondly, to evaluate the maximum OF, one has to estimate the OF generated by each of the tunnel surfaces, i.e., the floor, the ceiling, and the left and right walls. In the horizontal plane, as the honeybees’ lateral position was unknown here, we assumed that the honeybees were centred in the narrow tunnel^4^, and the OF generated by one wall would therefore be the unilateral OF and would depend directly on the forward speed, which did not vary significantly, as established immediately above. In the vertical plane, as reported above, each of the OFs generated by the nearest surface in the vertical plane (the ventral OF generated by the floor and the dorsal OF generated by the ceiling) were held constant by honeybees in the constant low-roof and “dorsal ditch” conditions in all the experiments despite the change in the entrance position (*E*_*Bottom*_ or *E* ^*Top*^) (see previous subsection and its statistical test). The level of the ventral OF maintained in the steady state was significantly greater than that of the dorsal OF (one-tailed two sample Wilcoxon-test *W* = 5250; $P\ll 0.001$, ***Pooled*** *ω*_*v*_: *ω*_*v*_(Fig. 1Bi 30–190 cm), *ω*_*v*_(Fig. 1Bii 30–190 cm), *ω*_*v*_(Fig. 2Bi 30–160 cm), *ω*_*v*_(Fig. 2Bii 30–160 cm), ***Pooled*** *ω* ^*d*^: *ω* ^*d*^(Fig. 1Ai 30–190 cm), *ω* ^*d*^(Fig. 1Aii 30–50 cm), *ω* ^*d*^(Fig. 1Aii 170–190 cm), *ω* ^*d*^(Fig. 2Ai 30–160 cm), *ω* ^*d*^(Fig. 2Aii 40–50 cm)).

In addition, when the floor was the nearest surface during the training step, the ventral and unilateral OFs did not differ significantly during the steady flight phase (two-sided *one sample Wilcoxon-test V* = 1230; *P* = 0.944; ***Pooled*** *ω*_*v*_: *ω*_*v*_(Fig. 1Bi 30–190 cm), *ω*_*v*_(Fig. 1Bii 30–190 cm), *ω*_*v*_(Fig. 2Bi 30–160 cm), *ω*_*v*_(Fig. 2Bii 30–160 cm); ***Pooled*** *ω*_*lateral*_: *ω*_*lateral*_(Fig. 1Bi 30–190 cm), *ω*_*lateral*_(Fig. 1Bii 30–190 cm), *ω*_*lateral*_(Fig. 2Bi 30–160 cm), *ω*_*lateral*_(Fig. 2Bii 30–160 cm)). When the ceiling was the nearest surface during the training step, the unilateral OF was significantly greater than the dorsal OF during steady flight (one-tailed *one sample Wilcoxon*-test *V* = 509; $P\ll 0.001$; ***Pooled*** *ω* ^*d*^: *ω* ^*d*^(Fig. 1Ai 30–190 cm), *ω* ^*d*^(Fig. 1Aii 30–50 cm), *ω* ^*d*^(Fig. 1Aii 170–190 cm), *ω* ^*d*^(Fig. 2Ai 30–160 cm), *ω* ^*d*^(Fig. 2Aii 40–50 cm); ***Pooled*** *ω* ^*lateral*^: *ω* ^*lateral*^(Fig. 1Ai 30–190 cm), *ω* ^*lateral*^(Fig. 1Aii 30–50 cm), *ω* ^*lateral*^(Fig. 1Aii 170–190 cm), *ω* ^*lateral*^(Fig. 2Ai 30–160 cm), *ω* ^*lateral*^(Fig. 2Aii 40–50 cm)). In conclusion, trained honeybees followed the ceiling although the translational OF generated by the ceiling was not the greatest OF, especially in comparison with the lateral OFs.

The present results are in line with the forward control loop hypothesis suggested by the ALIS model, but not entirely with the idea that a positioning control loop may be involved, as trained honeybees were found here to follow the ceiling even when this surface did not generate the greatest OF. When following the ceiling, trained honeybees may also take the unilateral OF into account. In addition, this OF maintained by the honeybees was not the greatest OF: two distinct OF magnitudes seem to be held constant in fact to ensure position control: either the side or ventral OF is maintained constant, and the dorsal OF is also maintained constant, whereas the ALIS model pointed to the use of a single positioning OF setpoint^15^. In order to account for these two discrepancies in the positioning control process with respect to the ALIS model^15^, the positioning control loop may involve the translational optic flow originating from several surfaces, - forming a complex OF pattern - in a much larger field of view than that previously thought to be involved.

### Joint OF-based learning and guidance

The present results clearly show that the visual stimuli learned and memorized by honeybees during the training step enabled them to perform floor and ceiling following behavior. Honeybees may in fact use visual cues such as the translational OF generated by several surfaces to guide themselves.

It was recently established that honeybees may use relative motion cues between landmarks and the background in a goal-localization task^25^. The latter authors explained the honeybees’ navigational performances in terms of a matching scheme including snapshots of a previously experienced OF pattern, such as that they referred to as the OF matching scheme. Based on the assumption that honeybees may store a snapshot of an OF pattern, they may locate the position of the reward by increasing the similarity between the current and memorized OF snapshot^25^. A probabilistic SLAM tool used in robotics failed, however, to show clearly whether or not bumblebees retrieved their position relative to an object near the nest using an OF pattern^26^. In their natural environment, honeybees are known to use landmarks such as trees and bushes to guide their flight toward a reward^27, 28^.

Based on these findings, it can be concluded that during the training session, honeybees must learn a more complex OF pattern than that previously predicted by the ALIS model. This complex OF pattern may be learned, memorized and matched with the currently perceived OF possibly involving a wide field of view including the ventral, dorsal or/and lateral regions of the insects’ compound eyes. In our opinion, these surfaces may generate an OF pattern that is memorized in a fairly wide field of view in terms of OF magnitudes and also in terms of the retinal positions of several local OF maxima.

By maintaining the similarity between the previously learned and the currently perceived OF patterns, trained honeybees were able to perform the tunnel-crossing task successfully in order to reach the reward.

All in all, the OFs perceived in the lateral, ventral and dorsal directions seem to be probably all involved in the honeybees’ altitude control system. The abundant draconian ecological constraints imposed on honeybees in their complex natural foraging environments including objects such as flowers, trees, foliage and/or bushes require these insects follow the surfaces forming by these bodies on the basis of dorsal, lateral and/or ventral OF cues.

## Methods

### Flight tunnel

The floor, roof and left wall of the flight tunnel used in this study consisted mainly of planks lined with red and white stripes. The right wall was made of thin white insect netting lined with stripes formed by a red gelatin filter (Lee Filters HT019), through which the honeybee’s flight paths could be recorded in the vertical plane. The flight tunnel was 220 cm long, 54 cm high and 25 cm wide at the entrance. The tunnel consisted of two parts: (i) the first half (part 1: from 0 cm to 110 cm) of the tunnel had a constant low roof with *H*_*tunnel*_ = 54*cm*, and (ii) the second half (part 2: from 110 cm to 220 cm) had a double roof consisting of a removable inner part set at a height of *H*_*tunnel*_ = 54 *cm*: when the inner roof was removed, the height of the second part of the tunnel was therefore *H*_*tunnel*_ = 100 *cm* (see Fig. 3). Depending on the experimental condition (constant low roof conditions or “dorsal ditch” conditions, respectively), the high-roofed tunnel was therefore either endowed with a uniform height (54 cm high) or increased to a height of 1 m in the second part (see the chronology of the procedure described in Supp. Fig. S1).

The tunnel was closed with a white plank at each end, and two manually operated openings (5 × 5 cm) placed either 5 cm from the roof or 5 cm from the ground gave each honeybee tested entry to the tunnel and access to the reward. The flight tunnel was placed outdoors and oriented to the North. It received no direct sunlight.

### Pattern

The pattern on the walls of the tunnel consisted of red and white stripes oriented transversely with respect to the bees’ flight paths (as in previous studies^8, 20^). Since honeybees are devoid of red-sensitive photoreceptors^29^, they perceive the red stripes as grey shades. These red stripes with two different widths (1 cm and 3 cm) formed a periodic 10 cm-wide pattern, as shown in Fig. 3. The angle subtended by the stripes ranged from 5.7° to 53° (a 1–10 cm pattern viewed from a distance of 10 cm) and from 0.5° to 5.3° (a 1–10 cm pattern viewed from a distance of 1 m). The Michelson contrast between the red and white stripes was *m* = 0.47 on the planks and *m* = 0.25 on the insect netting. Contrast was measured using a photodiode equipped with a green band-pass filter (Kodak Wratten N° 61), the transmission spectrum of which closely matched the spectral sensitivity of the honeybees’ green photoreceptors^29^, which are the receptors involved in motion vision^30–33^. A red filter placed in front of the camera monitoring the honeybees’ trajectories through the insect netting was used to optimize the contrast between the honeybees and the background.

### Experimental procedure

During the experimental period, a small set of four to six freely flying honeybees (*Apis mellifera*) were color-marked and trained daily to enter and fly alone along the outdoor tunnel to collect a sugar solution reward at the opposite end. Their flight path toward the reward was recorded with the digital camera from the insect-netting side, strictly in keeping with the chronology of the procedure depicted in Supplemental Fig. S1 using a group of different honeybees for each of the experiments (one group per experiment):

**Step 0** (***training: about 30 repeated flights***) We first trained a group of honeybees during about 30 flights to travel along a high-roofed tunnel endowed with a uniform height (called constant low-roof condition) to collect nectar in a reward box; the reward entrance was closed so that no visual cues about the position of the reward were available; after the honeybees had completed their trajectory, the door was opened to reward them at each flight).

**Step i** (***video-recording: 1 flight***) Immediately after the training step, the trajectory of same honeybee individuals was video-recorded under the same constant low-roof conditions; the reward entrance was again closed so that no visual cues about the position of the reward were available).

**Step ii** (***video-recording: 1 flight***) Immediately after the video-recording of the honeybees was filmed under low-roof conditions, the inner roof was removed and again, the trajectory of same honeybee individuals was recorded under “dorsal ditch” conditions; the reward entrance was again closed so that no visual cues about the position of the reward were available.

The following four experiments were carried out on 4 distinct groups of honeybees, as shown in Supplemental Fig. S2:

**Experiment 1** (Fig. 1A) **Step 0:** *E* ^*Top*^ → *R* ^*Top*^ **; Steps i & ii:** *E* ^*Top*^ The first group of honeybees (*n* = 22) were trained to enter the tunnel via the entrance located near the low roof under low roof conditions, before being released via the same entrance under “dorsal ditch” conditions.

**Experiment 2** (Fig. 1B) **Step 0:** *E*_*Bottom*_ → *R*_*Bottom*_ **; Steps i & ii:** *E*_*Bottom*_ The second group of honeybees (*n* = 24) were trained to enter the tunnel via the entrance located near the floor under low roof conditions before being released via the same entrance under “dorsal ditch” conditions.

**Experiment 3** (Fig. 2A) **Step 0:** *E* ^*Top*^ → *R* ^*Top*^ **; Step i:** *E* ^*Top*^ **; Step ii:** *E*_*Bottom*_ The third group of honeybees (*n* = 12) were trained to enter the tunnel via the entrance located near the low roof under low roof conditions before being released at the other entrance near the floor under “dorsal ditch” conditions.

**Experiment 4** (Fig. 2B) **Step 0:** *E*_*Bottom*_ → *R*_*Bottom*_ **; Step i:** *E*_*Bottom*_ **; Step ii:** *E* ^*Top*^ The fourth group of honeybees (*n* = 11) were trained to enter the tunnel via the entrance located near the floor under low roof conditions before being released via the entrance located near the low roof under “dorsal ditch” conditions.

The lower and upper entrances were both tagged on the outside with the same chequerboard pattern to facilitate the process of entrance detection for the honeybees. In order to incite the honeybees to enter the tunnel via the other entrance from that used during the training step, the training entrance was hidden and only the other entrance was tagged.

Only one honeybee at a time was allowed to enter the tunnel during each recording session. The digital camera was triggered at the moment when the honeybee entered the tunnel. During the recordings, the white door giving access to the reward remained seamlessly closed to rule out the presence of any undesirable attractive cues.

### Video recordings and flight path analysis

The honeybees’ trajectories were filmed at a rate of 20 frames per second with a high-resolution black-and-white CMOS camera (Prosilica EC1280). The camera was placed sideways, 2.3 m from the insect netting. The field of view (160 cm in width, 100 cm in height) covered the whole height of the tunnel, from abscissa *x* = 30 cm to abscissa *x* = 190 cm in the first set of experiments, and from abscissa *x* = 0 to abscissa *x* = 160 cm in the second set of experiments. Image sequences were processed and analysed using a custom-made Matlab program. This program automatically determined the honeybees’ flight height *h* in each frame as a function of the abscissa *x* along the tunnel axis so that the honeybee’s trajectory in the vertical plane could be plotted. No image correction was required because the objective was not a fish-eye and honeybees were not located in the corners of the frames.

### Statistical analysis

All the data recorded were included in the statistical analysis without removing any outliers. The flight parameters values recorded were computed for each tr﻿ajectory during the flight phase in which the insects showed stable flight patterns (also called *steady*-*state*), depending on the experimental condition, in order to prevent transient effects from being included in the computations.

Statistical data analyses were performed with the ‘R’ software program (http://www.r-project.org/). A few dataset do not exhibit a normal distribution. As a consequence, median and Median Absolute Deviation (Median ± MAD) values were computed for all dataset. Linear mixed model analyses^34^ using the *lme* function in R (R Foundation for Statistical Computing) were developed to test for the influence of repeated flight as well as of including multiple flight condition from individual honeybees. For height, distance, speed and optic flow analyses of the results and discussion sections, (i) the effects of the covariates (between individuals and experimental conditions factors) were not significant and were therefore not considered in the final analyses, and (ii) the variation between flights of the same bee was found to be similar to the variation of flights of different bees, indicating that each flight represented, in effect, an independent data point. *Wilcoxon*-tests (or *anova*.*lme*) were used to compare (or to assess likelihood) the median values of two (or more) distance, height, speed or optic flow distributions. Significant differences were determined with a significance level of *α* = 0.05. OF median values were pooled to be compared after their likelihood was check using *anova*.*lme*.

For speed and optic flow, all the existing datasets were used except for the experiment when honeybees reached a greater average ratio between their ascent speed and forward speed than in other experiments.

In Figs 1 and 2(iii–vi), the data distributions are presented using classical boxplots showing the median (red line), the 25th and the 75th percentiles (blue box), and the minimum and maximum values (black whiskers). The honeybees’ speed was calculated at each point on each honeybee’s trajectory, using a four-point derivative smoothing filter as follows: *v*_*x*_(*n*) = (2*x*(*n* − 2) + *x*(*n* − 1) + *x*(*n* + 1) + 2*x*(*n* + 2))/(10*Ts*). The honeybees’ forward speed *v*_*x*_ given below for each experiments are the median values of individual bees’ median speeds in each flight. The optic flow was computed using the ratio *v*_*x*_/*D*, where *D* is the distance from the tunnel surface: *D*_*side*_ = 12.5 *cm*, *D*_*floor*_ = *h*, *D*_*ceiling*_ = *H*_*tunnel*_ − *h*.

## Electronic supplementary material

Supp Data

## References

[1] Kennedy JS (1951). The migration of the desert locust (*schistocerca gregaria forsk*.). Phil. Trans. Royal Soc. B.

[2] David C (1979). Height control by free-flying drosophila. Physiol Entomol.

[3] David C (1982). Compensation for height in the control of groundspeed by drosophila in a new “barber’s pole” wind tunnel. J Comp Physiol A.

[4] Srinivasan MV, Zhang S, Lehrer M, Collett T (1996). Honeybee navigation en route to the goal: visual flight control and odometry. J Exp Biol.

[5] Srinivasan MV, Zhang S, Chahl J, Barth E, Venkatesh S (2000). How honeybees make grazing landings on flat surfaces. Biol Cybern.

[6] Baird E, Srinivasan MV, Zhang S, Cowling A (2005). Visual control of flight speed in honeybees. J Exp Biol.

[7] Baird E, Srinivasan MV, Zhang S, Lamont R, Cowling A (2006). Visual control of flight speed and height in honeybee. LNAI.

[8] Portelli G, Ruffier F, Franceschini N (2010). Honeybees change their height to restore their optic flow. J Comp Physiol A.

[9] Straw AD, Lee S, Dickinson MH (2010). Visual control of altitude in flying *drosophila*. Curr Biol.

[10] Linander N, Baird E, Dacke M (2016). Bumblebee flight performance in environments of different proximity. Journal of Comparative Physiology A.

[11] Nakayama K, Loomis J (1974). Optical velocity patterns, velocity-sensitive neurons, and space perception: a hypothesis. Perception.

[12] Koenderink JJ, van Doorn AJ (1987). Facts on optic flow. Biol Cybern.

[13] Serres JR, Masson GP, Ruffier F, Franceschini N (2008). A bee in the corridor: centering and wall-following. Naturwissenschaften.

[14] Franceschini N, Ruffier F, Serres J (2007). A bio-inspired flying robot sheds light on insect piloting abilities. Current Biology.

[15] Portelli G, Serres J, Ruffier F, Franceschini N (2010). Modelling honeybee visual guidance in a 3-d environment. J Physiol Paris.

[16] Ruffier F, Franceschini N (2005). Optic flow regulation: the key to aircraft automatic guidance. Robotics and Autonomous Systems.

[17] Ruffier F, Franceschini N (2015). Optic flow regulation in unsteady environments: A tethered MAV achieves terrain following and targeted landing over a moving platform. Journal of Intelligent & Robotic Systems.

[18] Expert F, Ruffier F (2015). Flying over uneven moving terrain based on optic-flow cues without any need for reference frames or accelerometers. Bioinspiration & Biomimetics.

[19] Serres J, Dray D, Ruffier F, Franceschini N (2008). A vision-based autopilot for a miniature air vehicle: joint speed control and lateral obstacle avoidance. Autonomous Robots.

[20] Portelli G, Ruffier F, Roubieu FL, Franceschini N (2011). Honeybees’ speed depends on dorsal as well as lateral, ventral and frontal optic flows. PloS one.

[21] Heusser D, Wehner R (2002). The visual centring response in desert ants, cataglyphis fortis. Journal of Experimental Biology.

[22] Duchon AP, Warren WH (2002). A visual equalization strategy for locomotor control: of honeybees, robots, and humans. Psychological Science.

[23] Vickers N, Baker T (1994). Visual feedback in the control of pheromone-mediated flight of *heliothis virescens* males (lepidoptera: Noctuidae). J Insect Behavior.

[24] Kirchner W, Srinivasan MV (1989). Freely flying honeybees use image motion to estimate object distance. Naturwissenschaften.

[25] Dittmar L, Stürzl W, Baird E, Boeddeker N, Egelhaaf M (2010). Goal seeking in honeybees: matching of optic flow snapshots?. J Exp Biol.

[26] Baddeley B (2009). What can be learnt from analysing insect orientation flights using probabilistic slam?. Biological Cybernetics.

[27] Collett TS, Rees JA (1997). View-based navigation in hymenoptera: multiple strategies of landmark guidance in the approach to a feeder. J Comp Physiol A.

[28] Fry S, Wehner R (2005). Look and turn: landmark-based goal navigation in honey bees. J Exp Biol.

[29] Menzel, R. & Backhaus, W. *Vision and visual dysfunction: the perception of color*, chap. Color vision in insects, 262–288 (Macmillan: London, 1991).

[30] Bishop LG (1970). The spectral sensitivity of motion detector units recorded in the optic lobe of the honeybee. J Comp Physiol A.

[31] Menzel R (1973). Spectral response of moving detecting and “sustaining” fibres in the optic lobe of the bee. J Comp Physiol A.

[32] Kaiser W, Liske E (1974). Die optomotorischen reaktionen von fixiert fliegenden bienen bei reizung mit spektrallichtern. J Comp Physiol A.

[33] Zhang S, Xiang W, Zili L, Srinivasan MV (1990). Visual tracking of moving targets by freely flying honeybees. Visual Neuroscience.

[34] McCulloch, C. E. & Neuhaus, J. M. *Generalized linear mixed models* (Wiley Online Library, 2001).

