# Supplementary material for: Altitude control in honeybees: joint vision-based learning and guidance
Source: Sci Rep. 2017 Aug 23;7:9231. doi: 10.1038/s41598-017-09112-5 (PMC5569062; doi:10.1038/s41598-017-09112-5)
Supplement: Supplementary file 1 — Supp Data [file 41598_2017_9112_MOESM1_ESM.pdf]

Altitude control in honeybees: joint vision-based learning and guidance

Geoffrey Portelli[1,2], Julien Serres[1], and Franck Ruffier[1,*]

[1]Aix Marseille Univ, CNRS, ISM, Marseille, France
[2]Université Côte d'Azur, CNRS, I3S, Sophia Antipolis, France
[*]franck.ruffier@univ-amu.fr

Supplemental Figure

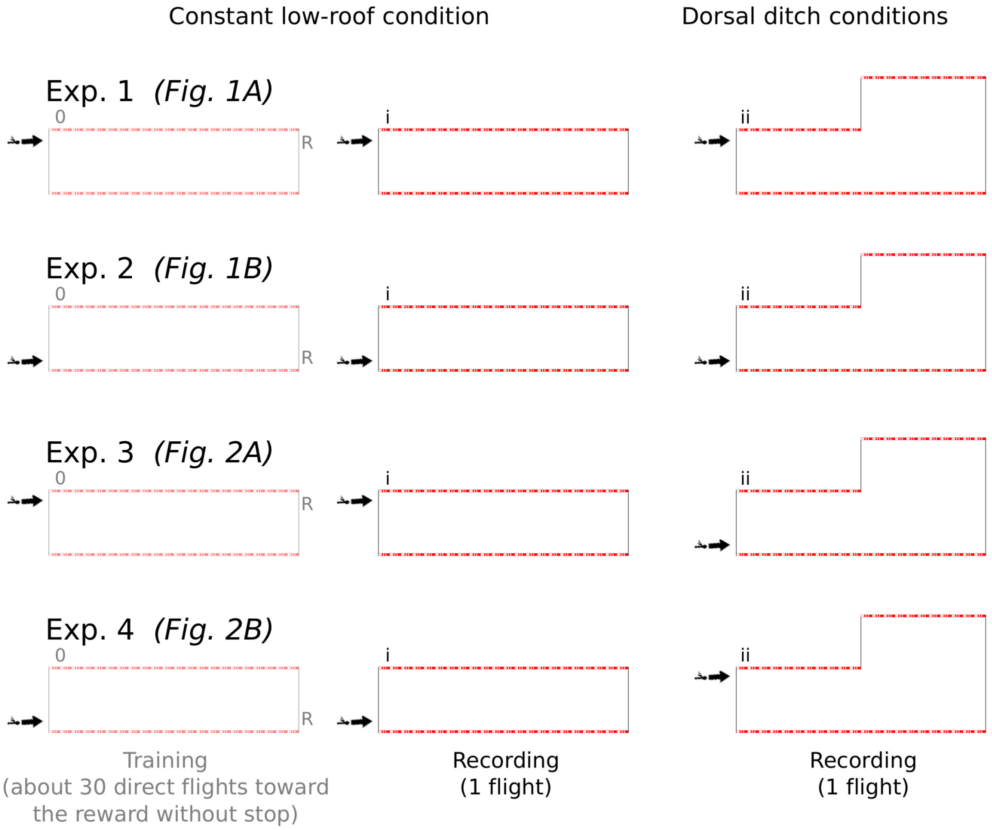

S.Fig. 1. Chronology of the procedure used in the 4 behavioural experiments. The same individual bees were trained and tested and their trajectories were recorded in line with the strict chronology of the steps.
Step 0 (the training step: about 30 flights) Honeybees were first trained during about 30 flights to travel along the tunnel endowed with a uniform height of 54 cm (called "Constant low-roof condition") to collect nectar in a reward box. They were rewarded at the end of each flight; the entrance to the reward box was closed during the flights so that no visual cues were available about the position of the reward.
Step i (video-recording: 1 flight) Immediately after the training step, individual bees' trajectories were recorded in this same tunnel endowed with a uniform height of 54 cm (called "Constant low-roof condition"); the entrance to the reward box was closed during the flights so that no visual cues were available about the position of the reward .
Step ii (video-recording: 1 flight) Immediately after recording the bees' trajectories under constant low-roof conditions, the inner roof was removed and the bees' trajectories were recorded in the presence of the "dorsal ditch" (called "Dorsal ditch conditions"); the entrance to the reward box was again closed during these flights so that no visual cues were available about the position of the reward.
Experiment 1 (Fig. 1A) Step 0: $E^{Top} \rightarrow R^{Top}$; Steps i & ii: E^{Top}.
The bees (n=22) were trained to enter the tunnel via the entrance placed near the low roof before being released near the low roof in the presence of the "dorsal ditch".
Experiment 2 (Fig. 1B) Step 0: $E_{Bottom} \rightarrow R_{Bottom}$; Steps i & ii: E_{Bottom}.
The bees (n=24) were trained to enter the tunnel via the entrance located near the floor before being released near the floor in the presence of the "dorsal ditch".
Experiment 3 (Fig. 2A) Step 0: $E^{Top} \rightarrow R^{Top}$; Step i: E^{Top}; Step ii: E_{Bottom}.
The bees (n=12) were trained to enter the tunnel via the entrance located near the low roof before being released near the floor in the presence of the "dorsal ditch".
Experiment 4 (Fig. 2B) Step 0: $E_{Bottom} \rightarrow R_{Bottom}$; Step i: E_{Bottom}; Step ii: E^{Top}.
The bees (n=11) were trained to enter the tunnel via the entrance located near the floor before being released near the low roof in the presence of the "dorsal ditch".
